# A Carrier Estimation Method Based on MLE and KF for Weak GNSS Signals

**DOI:** 10.3390/s17071468

**Published:** 2017-06-22

**Authors:** Hongyang Zhang, Luping Xu, Bo Yan, Hua Zhang, Liyan Luo

**Affiliations:** 1 School of Aerospace Science and Technology, Xidian University, Xi’an 710126, China; zhanghongyang@stu.xidian.edu.cn (H.Z.); BoYan@stu.xidian.edu.cn (B.Y.); zhanghua@mail.xidian.edu.cn (H.Z.); 2 School of Information and Communication, Guilin University of Electronic Technology, Guilin 541004, China; xiaoyan12027@163.com

**Keywords:** GNSS receiver, High sensitivity, MLE, KF

## Abstract

Maximum likelihood estimation (MLE) has been researched for some acquisition and tracking applications of global navigation satellite system (GNSS) receivers and shows high performance. However, all current methods are derived and operated based on the sampling data, which results in a large computation burden. This paper proposes a low-complexity MLE carrier tracking loop for weak GNSS signals which processes the coherent integration results instead of the sampling data. First, the cost function of the MLE of signal parameters such as signal amplitude, carrier phase, and Doppler frequency are used to derive a MLE discriminator function. The optimal value of the cost function is searched by an efficient Levenberg–Marquardt (LM) method iteratively. Its performance including Cramér–Rao bound (CRB), dynamic characteristics and computation burden are analyzed by numerical techniques. Second, an adaptive Kalman filter is designed for the MLE discriminator to obtain smooth estimates of carrier phase and frequency. The performance of the proposed loop, in terms of sensitivity, accuracy and bit error rate, is compared with conventional methods by Monte Carlo (MC) simulations both in pedestrian-level and vehicle-level dynamic circumstances. Finally, an optimal loop which combines the proposed method and conventional method is designed to achieve the optimal performance both in weak and strong signal circumstances.

## 1. Introduction

Global positioning systems (GPS) have been widely used in various fields. Whenever and wherever four or more satellites are visible, we can use GPS receivers to achieve positioning and navigation. The receiver can only capture and track the received signal from satellites which have an intensity above a certain power level. The signal intensity is usually referred to as signal-to-noise ratio (SNR) or carrier-to-noise spectral density ratio (*C*/*N*_0_). However, it is not always possible to ensure high *C*/*N*_0_ in some harsh circumstances such as in urban, forest and indoor areas. Since the carrier tracking loop is always the weakest link in a stand-alone GPS receiver, its threshold characterizes the unaided GPS receiver performance [1].

For weak signal applications, the two important factors that affect the performance of the receiver are the signal strength and dynamics of the receiver. The most commonly used loop, the phase-locked loop (PLL), has low sensitivity [1]. Increasing the integration time is a common solution to improve the sensitivity of receivers, such as coherent integration, non-coherent integration and differential integration [2,3]. However, increasing the integration time will reduce the dynamic performance of the phase-locked loop (PLL). The frequency-locked loop (FLL) is another common loop which has higher sensitivity and better dynamic performance. However, its accuracy is low. In urban and indoor circumstances, the satellite signal is severely attenuated. Received signal intensity is typically 10–30 dB lower than actual received power level in open environments, which brings a serious challenge to the conventional loops [4].

The maximum likelihood estimation (MLE) approach is known to yield optimal performance for GNSS signal tracking. Some loops based on MLE have been researched and applied in some specific signal environments [5,6,7,8,9,10,11]. Hurd et al. [5] proposed an approximate maximum likelihood method to track Doppler frequency and code delay for a high-dynamic receiver. The multipath signal parameters are estimated by MLE in [6,7]. Iterative and non-iterative MLE for Doppler frequency and code delay are discussed in [8,9]. Joint maximum likelihood estimation for position is proposed in [10] and its Cramér–Rao bound (CRB) is derived in [11]. These methods can achieve high performance in some specific environments. However, all of these methods process the sampling data (intermediate frequency, IF, raw samples) directly and perform complex processing or iteration operations. This is because the sampling data has a high data rate from 5 MHz up to 30 MHz. That implies a large computation burden and means more hardware or software resources are needed, which is undesirable. 

This paper proposes a low-complexity MLE carrier estimation method. In contrast to the existing methods, this method processes the coherent integration results instead of sampling data. It therefore has a small computation burden because the processed data rate is reduced from 5.714 MHz (the sampling rate) to 1000 Hz. The main contributions of this paper include two key points which are included in Section 3 and Section 4, respectively. In Section 3, the MLE discriminator is presented under the assumption that the pseudo random code (PRN) is stripped off. Its cost function with respect to the carrier phase, frequency and amplitude is given in Section 3.1. An efficient parameter estimation method by Levenberg–Marquardt (LM) algorithm is described in Section 3.2. Then, the performance of an MLE discriminator including CRB, dynamic performance and computation cost is analyzed in Section 3.3, Section 3.4 and Section 3.5, respectively. In Section 4, an adaptive Kalman filter (KF) is designed to improve the performance of the MLE discriminator. The basic equations of KF are given in Section 4.1 including the observation equation, state transition equation and their covariance matrix. It uses the amplitude estimate to adjust the observation noise matrix adaptively, so it is an adaptive KF. In Section 4.2, the block diagram of the proposed loop is given and illustrated in detail. In Section 5, some simulations are undertaken to demonstrate the performance of the proposed method. The performance of the LM method and KF is tested in Section 5.1 and Section 5.2, respectively. Monte Carlo (MC) simulations are made to calculate the tracking probability, tracking accuracy and bit error rate. A conventional FLL-assisted PLL is simulated too in order to make a comparison. Section 5.4 designs an optimal loop which combines the proposed loop and FLL-assisted PLL to achieve the optimal performance in weak and strong signal environments. 

## 2. Signal Model

The radio frequency (RF) signal transmitted by satellites is received by the receiver antenna. Then it is down-converted and digitized into the discrete intermediate frequency (IF) signal. The IF signal is sent to the baseband processing section to perform acquisition, tracking and demodulation. The acquisition process estimates coarse code phase and Doppler frequency which are used to initialize the tracking loop. Based on the acquisition results, the tracking loop can converge to the true values gradually and output the precise estimates of the code phase and Doppler frequency.

The IF signal can be expressed as data bits *D* modulated by pseudo-random code (PRN) *C* and carrier. With the sampling interval of *t_s_*, the discrete IF signal can be written as,

(1) $$r_{IF}[i]=a(it_{s}-\tau )\cdot D(it_{s}-\tau )\cdot C(it_{s}-\tau )\cos\left(2\pi \left(f_{IF}+f_{d}\right)it_{s}+\varphi_{0}\right)+w(it_{s}-\tau )$$

where *i* is the index of sampling points, *a* is the IF signal amplitude, τ is the code propagation delay, *f_IF_* and *f_d_* denote the intermediate frequency and Doppler frequency, respectively, *φ*_0_ is the initial carrier phase, and *w* is the noise term which is the white Gaussian noise.

**Figure 1:**
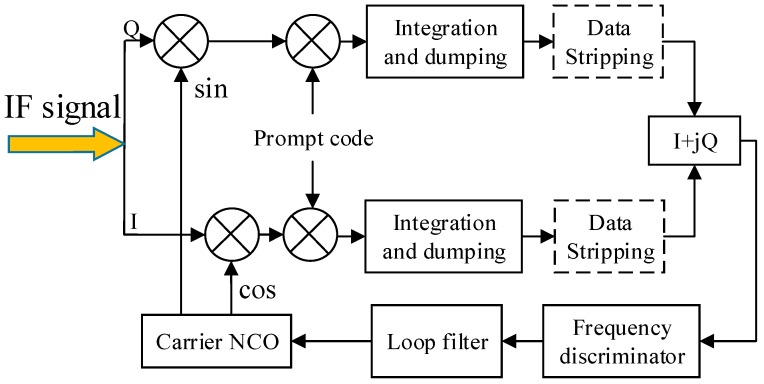
Block diagram of the conventional carrier tracking loop. NCO: numerically-controlled oscillator; IF: intermediate frequency.

For the general in-phase and quadrature (I/Q) processing method, IF signal performs frequency mixing with two local carriers which have a phase difference of 90 degrees, i.e., the sine and cosine carrier. Figure 1 shows the block diagram of the carrier tracking loop. After frequency mixing, code stripping is performed by multiplication with the local code. Then, coherent integration is performed to get a sequence of integration results. The integration process is operated under the assumption that the carrier is tracked accurately. Therefore, the carrier phase over the short integration time can be considered constant. Data bits must be removed if the integration time is more than the data length (20 ms for GPS L1C/A signal). In the following derivation, the integration time is no more than 20 ms, and PRN is assumed to be stripped off by the conventional delay-locked loop (DLL) completely. This assumption is reasonable because the proposed loop can replace the conventional PLL or FLL absolutely which will be explained in Section 4.2. The signal parameters of interest (i.e., signal amplitude, Doppler frequency and carrier phase) change slowly enough that they may reasonably be treated as unknown constants over the integration time. Then, the integration results of two channels can be written as a complex form [12],

(2) $$r[n]=A[n]\cdot D[n]\cdot \mathrm{sinc}(fT)\exp\left(j(2\pi fnT+\varphi )\right)+w_{ci}[n]$$

where *n* is the index of the integration results, *T* is the coherent integration time, *A*, *f* and *φ* are the amplitude, frequency and phase residual, respectively, and *w_ci_* is the complex noise term which is the accumulation of *w* in Equation (1). Because *w* is white Gauss noise, *w_ci_* is white Gauss noise too. In general, the noise in the I and Q channels is independent and has the same power. Therefore, the real part and imaginary part of *w_ci_* are irrelevant and have the same variance σ^2^. 

In the tracking process, *f* and *T* are small and sinc(*fT*) is approximately equal to 1 (for *T* = 1 ms and *f* = 10 Hz, sinc(*fT*) = 0.9998). Then Equation (2) can be rewritten as Equation (3).

(3) $$r[n]\approx A[n]\cdot D[n]\cdot \exp\left(j(2\pi fnT+\varphi )\right)+w_{ci}[n]$$

The integration results in Equation (3) are sent to the frequency discriminator and loop filter to get the frequency or phase residual estimate. The estimated residual is used to adjust the frequency and phase of the carrier numerically-controlled oscillator (NCO).

## 3. MLE Discriminator

### 3.1. Cost Function of MLE

Data bits in (3) may lead to 180-degree carrier-phase reversals. Therefore, there is a phase ambiguity of π between different data bit periods. The method to estimate data bit reversals will be discussed in Section 4.2 and is not considered in this section. Therefore, there are three unknown parameters *A*, *f* and *φ* in (3). To estimate them by MLE, the cost function must be derived first. 

Under the assumption of white Gaussian noise, the joint probability density function of *N* consecutive integration results can be expressed as (4) [13].

(4) $$p(V_{N}|A,f,\varphi )=\frac{1}{{\left(2\pi \sigma^{2}\right)}^{N}}\exp\left(-\frac{1}{2\sigma^{2}}\left(V_{N}-{\hat{V}}_{N}\right)W{\left(V_{N}-{\hat{V}}_{N}\right)}^{H}\right)$$

where $V_{N}=\left[r[0],r[1],...,r[N-1]\right]$ represents *N* coherent integration results, ${\hat{V}}_{N}$ is the estimate of *V_N_* in the absence of noise, *W* is a diagonal matrix and denotes the weighting factor of *r*[*n*], (*)*^H^* denotes the transpose and conjugate operation.

The estimates of the signal parameters *A*, *f* and *φ* are obtained by maximizing (4) which can achieve a minimum variance unbiased estimate with the Cramer–Rao lower bound (CRLB) [14]. Setting all the diagonal elements of *W* to 1, the log-likelihood cost function for various signal parameters is:

(5) $$\begin{array}{ll} \Lambda (\theta |r_{N}) & =-\frac{1}{2\sigma^{2}}\left(V_{N}-{\hat{V}}_{N}\right){\left(V_{N}-{\hat{V}}_{N}\right)}^{H}-N\ln\left(2\pi \sigma^{2}\right)\\ & =-\frac{1}{2\sigma^{2}}{\left|V_{N}-{\hat{V}}_{N}\right|}^{2}-N\ln\left(2\pi \sigma^{2}\right)\\ & =-\frac{1}{2\sigma^{2}}\sum_{n=0}^{N-1}{\left(R(r_{n})-A\cos(\alpha )\right)}^{2}-\frac{1}{2\sigma^{2}}\sum_{n=0}^{N-1}{\left(I(r_{n})-A\sin(\alpha )\right)}^{2}-N\ln\left(2\pi \sigma^{2}\right)\\ & =-\frac{1}{2\sigma^{2}}\sum_{n=0}^{N-1}\left\{{\left|r_{n}\right|}^{2}+A^{2}-2A\left[R(r_{n})\cdot \cos(\alpha )+I(r_{n})\cdot \sin(\alpha )\right]\right\}-N\ln\left(2\pi \sigma^{2}\right) \end{array}$$

where α=2πfnT+φ, $r_{n}=r[n]$ is used to simplify the expression, $\theta ={\left[\begin{array}{ccc} A & f & \varphi \end{array}\right]}^{T}$ represents the signal parameter vector, *R*(*) and *I*(*) denote the real and imaginary part, respectively. 

The MLE of the signal parameters can be obtained by maximizing the log-likelihood cost function in (5), based on the observation vector $r_{N}$ satisfying:

(6) $$\frac{\partial \Lambda (\theta |r_{N})}{\partial \theta }=0$$

The partial derivative for *A* in (6) is easy to calculate.

(7) $$\frac{\partial \Lambda }{\partial A}=-\frac{1}{\sigma^{2}}\sum_{n=0}^{N-1}\left\{A-\left[R(r_{n})\cdot \cos(\alpha )+I(r_{n})\cdot \sin(\alpha )\right]\right\}$$

Then, *A* can be estimated by setting the partial derivative (7) to zero.

(8) $$A=\frac{1}{N}\sum_{n=0}^{N-1}\left[R(r_{n})\cdot \cos(\alpha )+I(r_{n})\cdot \sin(\alpha )\right]$$

In (5), the term |*r_n_*|^2^ on the right-hand side can be treated as a constant (no information pertains to the signal parameters). The component *A^2^* contains only the parameter *A*. Thus, the partial derivatives of Λ for *f* and *φ* depend only on the third component in which *A* can be regarded as a constant coefficient. Taking no account of *A* and ignoring irrelevant terms, the new cost function for *f* and *φ* can be written as:

(9) $$L=\sum_{n=0}^{N-1}\left[R(r_{n})\cdot \cos(\alpha )+I(r_{n})\cdot \sin(\alpha )\right]$$

**Figure 2:**
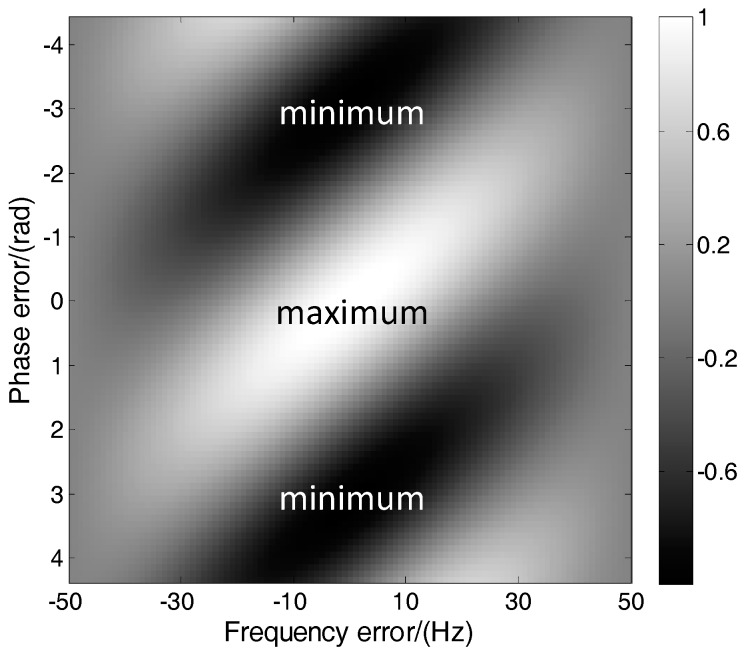
Normalized maximum likelihood estimation (MLE) cost function as a function of the frequency and phase errors.

Therefore, the MLE is converted to a two-dimensional optimization problem to search *f* and *φ* which maximize *L*. Figure 2 shows the normalized cost function in (9) with respect to the frequency and phase errors. The integration time is 1 ms and *N* is 20. It can be seen that the cost function reaches the maximum value when the Doppler frequency and phase errors are zero. It is a periodic function of the phase and has symbol flipping when the phase changes π. This is easy to demonstrate by (9). Therefore, the frequency and phase can be obtained by searching the maximum or minimum points of the cost function.

### 3.2. Parameters Estimation by LM Algorithm

Searching the extreme value of the cost function in (9) is a two-dimensional optimization problem. A simple method, known as the grid search method, is to search the two-dimensional space step by step. This method is easy to implement and the search scope can be adjusted arbitrarily. However, its precision is limited by the resolution. Improving the resolution will increase the computation burden significantly. 

Another method is the gradient-based method which searches the extreme point along the gradient direction. The Levenberg–Marquardt algorithm, also known as the damped least-squares (DLS) method, is one of the most effective optimization methods to solve non-linear least squares problems [14]. It can be seen as an improved Gauss–Newton (GN) algorithm and converges to the optimal value iteratively. It has high efficiency and is not limited by the resolution. Its core formula to search the maximum can be expressed as:

(10) $${\hat{\theta }}_{i+1}={\hat{\theta }}_{i}+{\left(H_{i}+d\right)}^{-1}G_{i},\;i=0,1,2...$$

where *i* represents the iteration index, and $\hat{\theta }$ is the 2-by-1 MLE state vector (*f* and *φ*). *d* is a 2-by-2 diagonal matrix which has two functions, ensuring that *H_i_+d* is a positive definite matrix and adjusting the convergence rate. *G_i_* and *H_i_* are the 2-by-1 gradient vector and the 2-by-2 pseudo-Hessian matrix respectively, defined as follows:

(11) $$G_{i}={\left[\frac{\partial L}{\partial \theta }\right]}_{\theta ={\hat{\theta }}_{i}}$$

(12) $$H_{i}={\left[\frac{\partial^{2}L}{\partial \theta^{2}}\right]}_{\theta ={\hat{\theta }}_{i}}$$

where,

(13) $$\frac{\partial L}{\partial \theta }={\left[\begin{array}{cc} \frac{\partial L}{\partial f} & \frac{\partial L}{\partial \varphi } \end{array}\right]}^{T}\;\mathrm{and}\;\frac{\partial^{2}L}{\partial \theta^{2}}=\left[\begin{array}{cc} \frac{\partial^{2}L}{\partial f^{2}} & \frac{\partial^{2}L}{\partial f\partial \varphi }\\ \frac{\partial^{2}L}{\partial \varphi \partial f} & \frac{\partial^{2}L}{\partial \varphi^{2}} \end{array}\right]$$

Detailed expressions of the terms in (13) are provided as follows:

(14) $$\left\{ \begin{array}{l} \frac{\partial L}{\partial f}=2\pi T\sum_{n=0}^{N-1}n\left(-R(r_{n})\cdot \sin(\alpha )+I(r_{n})\cdot \cos(\alpha )\right)\\ \frac{\partial L}{\partial \varphi }=\sum_{n=0}^{N-1}\left(-R(r_{n})\cdot \sin(\alpha )+I(r_{n})\cdot \cos(\alpha )\right)\\ \frac{\partial L}{\partial f^{2}}=-4\pi^{2}T^{2}\sum_{n=0}^{N-1}n^{2}\left(R(r_{n})\cdot \cos(\alpha )+I(r_{n})\cdot \sin(\alpha )\right)\\ \frac{\partial L}{\partial \varphi^{2}}=-\sum_{n=0}^{N-1}\left(R(r_{n})\cdot \cos(\alpha )+I(r_{n})\cdot \sin(\alpha )\right)\\ \frac{\partial L}{\partial f\partial \varphi }=\frac{\partial L}{\partial \varphi \partial f}=-2\pi T\sum_{n=0}^{N-1}n\left(R(r_{n})\cdot \cos(\alpha )+I(r_{n})\cdot \sin(\alpha )\right) \end{array}\right.$$

**Figure 3:**
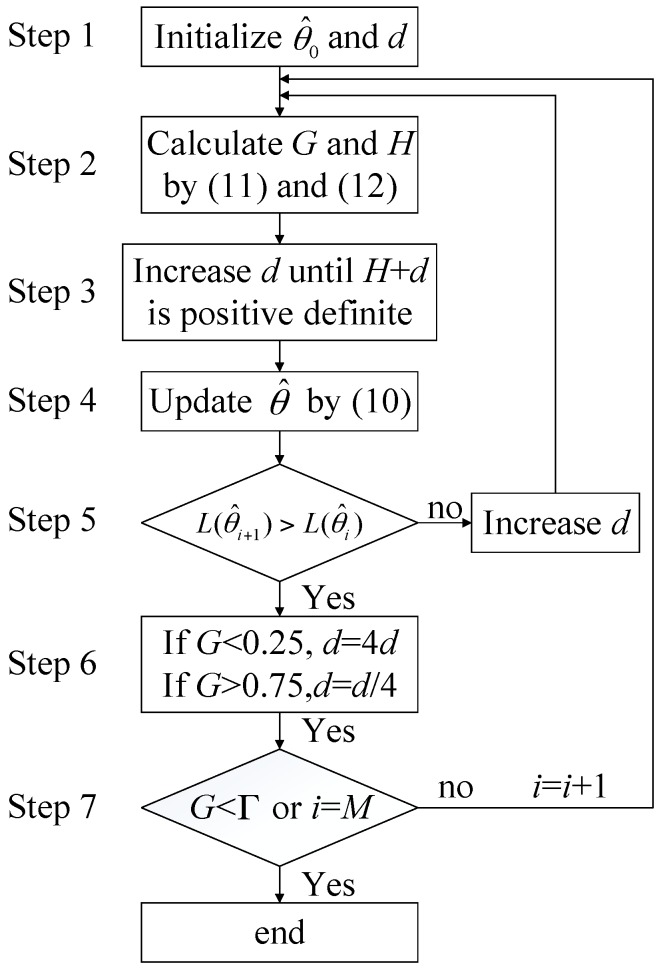
Block diagram of the LM algorithm. LM: Levenberg–Marquardt.

The detailed realization of the LM algorithm is shown in Figure 3. First, the initial values for ${\hat{\theta }}_{0}$ and *d* are specified in Step 1. In the tracking process, the phase and frequency are assumed to track accurately. Therefore, ${\hat{\theta }}_{0}={\left[0\;0\right]}^{T}$. *d* is initialized as an experience value. Step 2 calculates gradient matrix *G* and Hessian matrix *H* by (11) and (12). Step 3 ensures that *H*+*d* is positive definite. If *H*+*d* is negative definite, *d* must be increased. This step is the main difference between the LM algorithm and GN algorithm. It ensures the inverse matrix of *H*+*d* is always present and the iteration process can continue. Step 4 updates the parameters matrix by (10). Based on this, the cost function values at ${\hat{\theta }}_{i}$ and ${\hat{\theta }}_{i+1}$ are calculated and compared in Step 5. In order to ensure efficient iteration, $L\left({\hat{\theta }}_{i+1}\right)$ must be larger than $L\left({\hat{\theta }}_{i}\right)$ (searching the maximum value). Otherwise, *d* must be increased again. Step 6 adjusts the convergence rate by increasing or decreasing *d*. When $\hat{\theta }$ is away from the extreme point (gradient values are large), *d* is decreased to achieve fast convergence. When $\hat{\theta }$ is close to the extreme point (gradient values are small), *d* is increased to achieve slow convergence. The inequality *G* < 0.25 denotes every element in *G* is less than 0.25, the same as below. Step 7 ends the iteration process when gradient is smaller than the threshold Γ or the iteration number exceeds the threshold *M*. 

### 3.3. Cramér–Rao Bound (CRB)

The CRB expresses a lower bound on the variance of estimators of a deterministic parameter. The derivation method of CRB is mature and can be found in [13]. Based on the work in [13], the discrete form of the CRB of the proposed maximum likelihood (ML) discriminator is derived in this section. The multiple-parameter CRB for any unbiased estimate of a generic, real-valued parameter vector *θ* means the covariance matrix of the estimates *C*(*θ*) is bounded as

(15) $$C(\hat{\theta })>J^{-1}(\theta )$$

where *J*(*θ*) is commonly referred to as the Fisher information matrix (FIM), whose inverse is the CRB matrix. With Λ(θ) being the log-likelihood function, the FIM elements are defined by:

(16) $${\left[J(\theta )\right]}_{u,v}=-E\left\{\frac{\partial^{2}\Lambda (\theta )}{\partial \theta_{u}\partial \theta_{v}}\right\}$$

where *u*, *v* denotes the index of the parameters.

The second-order partial derivative in (16) can be calculated as,

(17) $$\left\{ \begin{array}{l} \frac{\partial^{2}\Lambda }{\partial f^{2}}=-\frac{4}{\sigma^{2}}A\pi^{2}T^{2}\sum_{n=0}^{N-1}n^{2}\left(R(r)\cdot \cos(\alpha )+I(r)\cdot \sin(\alpha )\right)\\ \frac{\partial^{2}\Lambda }{\partial \varphi^{2}}=-\frac{A}{\sigma^{2}}\sum_{n=0}^{N-1}\left(R(r)\cdot \cos(\alpha )+I(r)\cdot \sin(\alpha )\right)\\ \frac{\partial^{2}\Lambda }{\partial f\partial \varphi }=\frac{\partial^{2}\Lambda }{\partial \varphi \partial f}=-\frac{2\pi TA}{\sigma^{2}}\sum_{n=0}^{N-1}n\left(R(r)\cdot \cos(\alpha )+I(r)\cdot \sin(\alpha )\right) \end{array}\right.$$

Substituting (17) to (16), the FIM can be obtained.

(18) $$J(\theta )=\frac{A^{2}}{\sigma^{2}}\left[\begin{array}{cc} N & \pi TN\left(N-1\right)\\ \pi TN\left(N-1\right) & 2\pi^{2}T^{2}\left(N-1\right)N\left(2N-1\right)/3 \end{array}\right]$$

Then CRB of *f* and *φ* can be expressed as,

(19) $$\left\{ \begin{array}{l} CR_{f}=1/{\left[J(\theta )\right]}_{1,1}=3\sigma^{2}/2A^{2}\pi^{2}T^{2}\left(N-1\right)N\left(2N-1\right)\\ CR_{\varphi }=1/{\left[J(\theta )\right]}_{2,2}=\sigma^{2}/A^{2}N \end{array}\right.$$

Similarly, the CRB of *A* is derived separately as,

(20) $$CR_{A}=-/E\left\{\frac{\partial^{2}\Lambda (\theta )}{\partial A^{2}}\right\}=\sigma^{2}/N$$

Equations (19) and (20) determine the lower bound on the variance of *f*, *φ* and *A*. As explained in Section 2, *A*^2^ and 2σ^2^ can be seen as the signal power and noise power of *r*[*n*], respectively. $A^{2}/2\sigma^{2}$ denotes the SNR of *r*[*n*] which is proportional to the integration time *T* when the RF signal power and sampling rate are fixed. Therefore, the CRB of *f* and *φ* can be seen as a constant multiplied by the term $1/T^{3}\left(N-1\right)N\left(2N-1\right)$ and 1/*TN*, respectively. It is obvious that increasing *N* and *T* will reduce the CRB of *f* and *φ*. In addition, increasing *N* and reducing *T* will reduce the CRB of *f* too when *NT* (the discriminator update time) is fixed. For example, the strategy *N* = 20, *T* = 1 ms can get lower CRB of *f* than the strategy *N* = 10, *T* = 2 ms. Given the *C/N*_0_ of IF signal, SNR of *r*[*n*] expressed in the form of dB can be calculated as:

(21) $$SNR=C_{N0}-10\log_{10}\left(BW\right)+10\log_{10}\left(f_{s}T\right)$$

where *C_N0_* is the *C*/*N*_0_ of IF signal expressed in the form of dB, and *BW* is the noise bandwidth of the receiver. The second term on the right side converts SNR to *C*/*N*_0_ and the third term on the right side denotes the coherent integration gain. 

**Figure 4:**
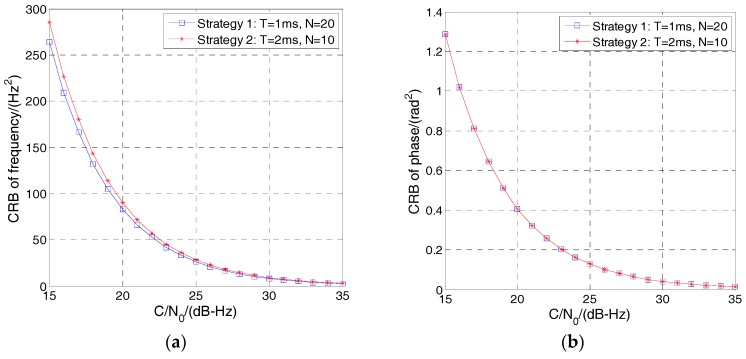
Cramér–Rao bound (CRB) of *f* and *φ* with respect to carrier-to-noise spectral density ratio (*C*/*N*_0_) of the IF signal. (**a**) CRB of *f*. (**b**) CRB of *φ*.

Figure 4 shows the CRB of *f* and *φ* with respect to *C*/*N*_0_ of IF signal. It can be seen that the CRB increases exponentially with the *C*/*N*_0_ decreases. Strategy 1 can obtain lower CRB of *f* than strategy 2. However, for the CRB of *φ*, there is no difference.

### 3.4. Dynamic Characteristics

The LM method is easy to converge to local optimal values if the initial point is not correct. This means the initial point must be in a pull-in range, i.e., the main peak of the cost function, in each update. The dynamic tolerance of the LM algorithm means the frequency change does not exceed half the width of the main peak during the update interval. 

Ignoring the noise term in (9), it can be simplified as:

(22) $$\begin{array}{ll} L_{s} & =\sum_{n=0}^{N-1}\left(\begin{array}{l} \cos\left(2\pi fnT+\varphi_{0}\right)\cdot \cos\left(2\pi (f+\Delta f)nT+\varphi_{0}+\Delta \varphi \right)\\ +\sin\left(2\pi fnT+\varphi_{0}\right)\cdot \sin\left(2\pi (f+\Delta f)nT+\varphi_{0}+\Delta \varphi \right) \end{array}\right)\\ & =\sum_{n=0}^{N-1}\cos\left(2\pi \Delta fnT+\Delta \varphi \right) \end{array}$$

where Δf and Δφ are the frequency and phase error, respectively.

The cost function in (22) is the accumulation of the cosine function whose phase is from Δφ to 2πΔf(N−1)T+Δφ. It is obvious that *Ls* reaches the minimum when 2πΔf(N−1)T+Δφ is equal to 3π/2 or −3π/2. Therefore, the main peak of L is in the range of $\left[\frac{-3\pi /2-\Delta \varphi }{2\pi NT},\frac{3\pi /2-\Delta \varphi }{2\pi NT}\right]$ which is inversely proportional to the discriminator update time and related to the phase error. 

**Figure 5:**
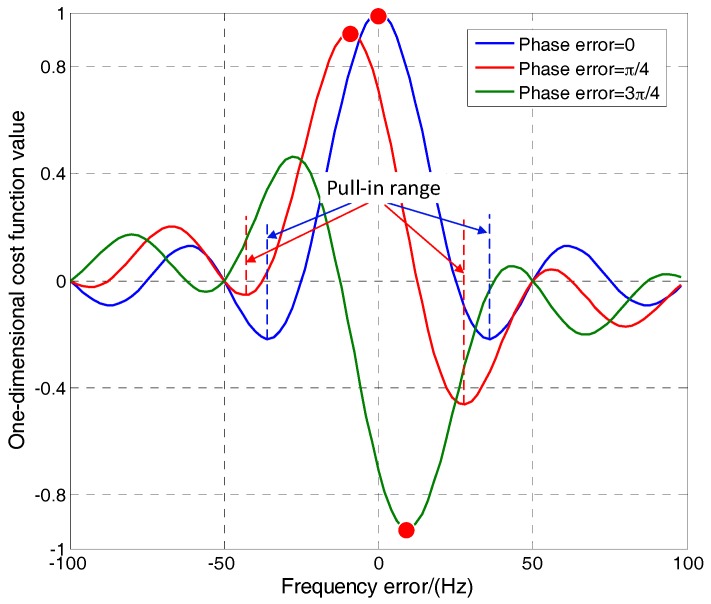
Cost function with respect to the frequency error and various phase errors.

Figure 5 shows the one-dimensional cost function in (22) with respect to the frequency error with various phase errors. The parameters are the same as those in Figure 3. It can be seen that the position of the extreme point (the red spot) changes with the phase error changing. When the phase error is more than π/2 or less than −π/2, the main peak becomes a negative peak. To avoid converging to local optimal values, the initial point of LM algorithm must be in the range of the main peak. Assuming the phase is tracked precisely (Δφ≈0), the pull-in range of LM algorithm is [−3/4*NT*, 3/4*NT*]. This means the tolerable Doppler rate is 3/4(*NT*)^2^ Hz/s which is inversely proportional to the square of the discriminator update time. Therefore, increasing the discriminator update time will lead to reduction in dynamic tolerance. However, to improve the sensitivity of the receiver, increasing the update time is necessary. It is a compromise between dynamic tolerance and sensitivity. 

When the discriminator update time is more than 20 ms, the integration results must be squared to remove the data bits. This will lead to two effects on the performance of the loop. Firstly, the Doppler frequency and phase residuals are doubled, resulting in halved dynamic tolerance. Secondly, the noise term is squared too, resulting in SNR loss (square loss) [15]. 

The SNR gain and dynamic tolerance should be considered simultaneously to choose the optimal discriminator update time. It is not difficult to find that (9) is equivalent to the coherent integration (NT≤20 ms) or non-coherent integration (NT>20 ms) when the Doppler frequency and phase residual are zero. The SNR gain at the maximum point of *L* is equal to coherent or non-coherent integration gain, which have been derived in [16]. 

Table 1 shows the dynamic tolerance and SNR gain of the proposed algorithm with respect to the different discriminator update time. The *C*/*N*_0_ of the received signal is 30 dB-Hz. It can be seen that the dynamic tolerance decreases rapidly with *NT* increasing. The SNR gain reaches to 76.1 dB when the discriminator update time is 20 ms. However, it drops to 72.9 dB when the discriminator update time increases from 20 ms to 40 ms because of the square loss. Until the discriminator update time reaches 100 ms, the SNR gain goes back to 76.9 dB with a low dynamic tolerance of 7.1 m/s^2^. So considering both the dynamic performance and SNR gain, 20 ms is chosen as the discriminator update time.

### 3.5. Computation Cost

In Figure 1, the basic frequency mixing and integration process can be implemented easily in the hardware circuit. However, the subsequent LM algorithm needs to be processed by the software. Compared with the existing methods [5,6,7,8,9,10,11], the proposed ML discriminator processes the coherent integration results instead of the sampling data. After the coherent integration process, the data rate is reduced from 1/*t_s_* to 1/*T*. Therefore, the amount of data is reduced by *T*/*t_s_* times. For the sampling rate of 5.714 MHz and the integration time of 1 ms, the amount of data is reduced by 5714 times. Therefore, the proposed method has a great improvement in efficiency compared with the existing MLE-based methods. 

In the LM algorithm, the main computation is the gradient and Hessian matrices, i.e., (14). Removing repeated calculation, and with the *n*^2^ term stored in advance, the actual calculation cost in an iteration is *N* sine and cosine, 6*N* multiplication and addition. Considering the maximum number of iterations is *M*, the total computation cost in an observation interval is about *MN* sine and cosine, 6*MN* multiplication and addition operation. Therefore, for *M* = 6, *N* = 20 and *T* = 1 ms, the processing requirements are 120 sine and cosine, 840 multiplication and addition operation of 20 ms, which are easy to implement. 

## 4. Adaptive Kalman Filter

### 4.1. Basic Equations of KF

After frequency and phase residual estimation by the MLE discriminator, a KF can be used to get fair estimates. KF is an optimal estimator by using the state space concept and system error model. The detailed process of KF has been documented clearly in [17]. Here, only the state transition equation and observation equation of KF is given.

The state vector is defined as $x_{k}={\left[\begin{array}{ccc} \varphi_{k} & w_{k} & {\dot{w}}_{k} \end{array}\right]}^{T}$. *k* is the index of the update period of the filter. $w_{k}=2\pi f_{k}$ is the angular frequency and ${\dot{w}}_{k}$ is the angular frequency rate. Authors of [18] have derived a state transition function whose state vector includes the code phase. Because the code tracking loop is not considered in this paper, the state transition function of the proposed KF is reproduced from [18] and modified by ignoring the code phase term. Its discrete form is expressed as:

(23) $$x_{k+1}=\left[\begin{array}{ccc} 1 & \Delta t & \frac{\Delta t^{2}}{2}\\ 0 & 1 & \Delta t\\ 0 & 0 & 1 \end{array}\right]x_{k}+\left[\begin{array}{ccc} w_{rf} & 0 & 0\\ 0 & w_{rf} & 0\\ 0 & 0 & \frac{w_{rf}}{c} \end{array}\right]\left[\begin{array}{c} w_{b}\\ w_{d}\\ w_{a} \end{array}\right]$$

where Δt=NT is the update interval of KF which is equal to the discriminator update time. The second term on the right side is the process noise term and has a covariance matrix *Q*. $w_{rf}$ is the angular frequency of the RF signal. $w_{b}$ and $w_{d}$ is the carrier phase noise and carrier frequency noise due to the local oscillator, respectively. $w_{a}$ is the carrier frequency rate noise. *Q* is given by (24).

(24) $$\begin{array}{ll} Q & =F\cdot E[W_{rf}\cdot {W_{rf}}^{T}]\cdot F^{T}\\ & ={\left(\frac{w_{rf}}{c}\right)}^{2}q_{a}\left[\begin{array}{ccc} \frac{\Delta t^{5}}{20} & \frac{\Delta t^{4}}{8} & \frac{\Delta t^{3}}{6}\\ \frac{\Delta t^{4}}{8} & \frac{\Delta t^{3}}{3} & \frac{\Delta t^{2}}{2}\\ \frac{\Delta t^{3}}{6} & \frac{\Delta t^{2}}{2} & \Delta t \end{array}\right]+{w_{rf}}^{2}q_{d}\left[\begin{array}{ccc} \frac{\Delta t^{3}}{3} & \frac{\Delta t^{2}}{2} & 0\\ \frac{\Delta t^{2}}{2} & \Delta t & 0\\ 0 & 0 & \Delta t \end{array}\right]+{w_{rf}}^{2}q_{b}\left[\begin{array}{ccc} \Delta t & 0 & 0\\ 0 & 0 & 0\\ 0 & 0 & 0 \end{array}\right] \end{array}$$

where *F* and *W_rf_* are the state transition matrix and observation noise matrix in (23) respectively, and $q_{b}$, $q_{d}$ and $q_{a}$ are the power spectral density of $w_{b}$, $w_{d}$ and $w_{a}$, respectively. Given h-parameters of the local oscillator, $q_{b}$ and $q_{d}$ can be calculated by:

(25) $$\left\{ \begin{array}{l} q_{b}=\frac{h_{0}}{2}\\ q_{d}=2\pi^{2}h_{-2} \end{array}\right.$$

Where $h_{0}$, $h_{-2}$ is the h-parameters of oscillator. For a voltage-controlled temperature-compensated oscillator (VCTCXO), $h_{0}=1\times 10^{-21}$ and $h_{-2}=1\times 10^{-20}$ is given in [18].

The MLE discriminator can output frequency and phase estimates. Choosing them as observations, the observation equation is written as,

(26) $$\left[\begin{array}{c} \hat{\varphi }\\ \hat{f} \end{array}\right]=\left[\begin{array}{ccc} 1 & 0 & 0\\ 0 & 1 & 0 \end{array}\right]x_{k}+v_{k}$$

where *v_k_* is the observation noise which has the covariance matrix *R_k_*. 

It is obvious that *R_k_* is the variance matrix of phase and frequency estimation errors. It can be derived as the inverse matrix of FIM [13].

(27) $$\begin{array}{ll} R_{k}=J^{-1}(\theta )= & \frac{\sigma^{2}}{A^{2}}\frac{3}{2\pi^{2}T^{2}(N-1)N(2N-1)-3\pi^{2}T^{2}{\left(N-1\right)}^{2}N}*\\ & \left[\begin{array}{cc} 2\pi^{2}T^{2}\left(N-1\right)\left(2N-1\right)/3 & -\pi T\left(N-1\right)\\ -\pi T\left(N-1\right) & 1 \end{array}\right] \end{array}$$

In (27), the term on the right side can be considered as $\sigma^{2}/A^{2}$ multiplied by a constant matrix when *T* and *N* is fixed. *A* can be estimated by (8). σ^2^ is estimated by,

(28) $$\sigma^{2}=\left(r_{N}-{\hat{r}}_{N}\right){\left(r_{N}-{\hat{r}}_{N}\right)}^{H}/2N$$

As explained above, $A^{2}/2\sigma^{2}$ is the SNR of integration result signal. Therefore, *R_k_* is adjusted adaptively by SNR estimation in each update. Meanwhile, *C*/*N*_0_ of the received signal can be estimated by (29).

(29) $$C/N_{0}=10\log_{10}\left(A^{2}/2\sigma^{2}\right)-10\log_{10}\left(f_{s}T\right)+10\log_{10}\left(BW\right)$$

### 4.2. ML-KF Loop

The KF needs to predict the information at the next moment using the estimated phase and frequency. However, data bit reversals will lead to 180-degree phase ambiguity. KF will output an incorrect result in this case. Therefore, data bits must be stripped off or estimated if KF is used. As illustrated above, the cost function value will change its symbol if the data reversal happens. This can be used to judge the data reversals. In the update epoch k, the cost function value at the initial point (*L*(*θ*_0_)) is calculated first. If *L*(*θ*_0_) > 0, the data bit is the same as the last bit and the phase does not need to be corrected. On the contrary, if *L*(*θ*_0_) < 0, the data reversal happens and the phase needs to be corrected by adding π. In this way, the data bits can be estimated.

**Figure 6:**
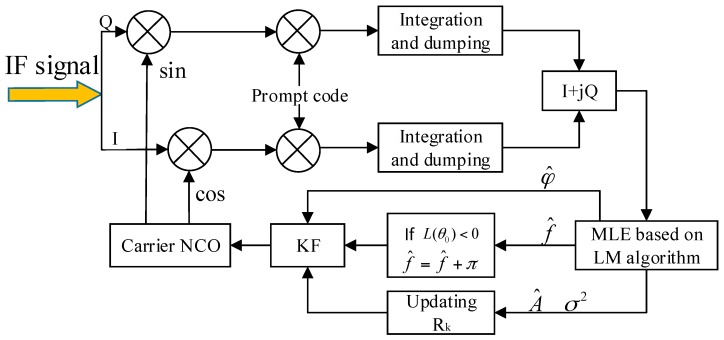
Block diagram of the ML-KF loop. ML-KF: maximum likelihood and Kalman filter.

The block diagram of the ML-KF loop is shown in Figure 6. The integration results are sent to the MLE estimator. The frequency and phase is estimated first by LM iteration processing. Then, $\hat{A}$ and σ^2^ are estimated by (8) and (28), respectively. They are used to update the observation noise covariance matrix *R_k_* and estimate *C*/*N*_0_ by (27) and (29), respectively. $\hat{f}$ is corrected by adding π if *L*(*θ*_0_) < 0. All these information is set to KF to get fair estimates of phase and frequency. Finally, the output of KF is used to adjust the frequency and phase of the carrier NCO.

In terms of the structure of the receiver, the ML-KF loop can be used to replace the traditional PLL or FLL absolutely. This is because they use the same input (the correlation outputs of the prompt branch) and output the same results (Doppler frequency and phase residuals) to adjust the carrier NCO. Therefore, a standard DLL architecture is compatible with the proposed method. Actually, they can be seen as two independent loops. Therefore, the perfect wipe off of the PRN can be assumed to avoid the influence of irrelevant factors.

## 5. Simulation Results

### 5.1. Simulation Results of MLE Discriminator

**Figure 7:**
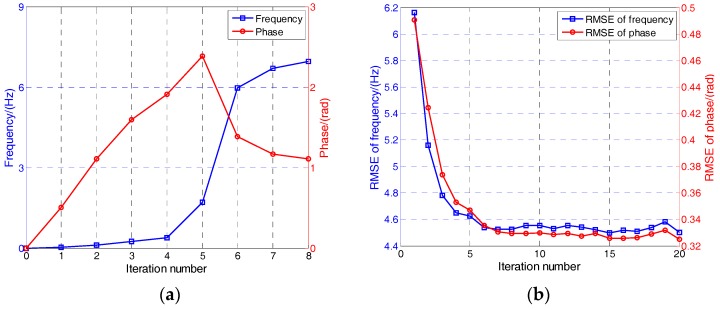
Convergence performance of the LM algorithm. (**a**) Frequency and phase convergence curves of a single example; (**b**) Root-mean-square error (RMSE) of frequency and phase with respect to the iteration number.

To test the validity of the proposed MLE discriminator, the convergence curves of phase and frequency are shown in Figure 7a. The simulation parameters are listed in Table 2. The true frequency and phase error are 7 Hz and 1 rad, respectively, expressed in coordinates as (7, 1). It can be seen that the iteration process ends after eight iterations, which means the gradient matrix G reaches the threshold. The frequency and phase converge to (6.9, 1.1) finally. The LM algorithm shows high efficiency and accuracy in this process. 

Figure 7b shows the root-mean-square error (RMSE) of the frequency and phase with respect to the number of iteration. The initial frequency residual is random from −10 to 10 Hz and the initial phase residual is random from −1 to 1 rad. In total, 50,000 MC simulations are made to calculate the RMSE. It can be seen that the accuracy of the frequency and phase have the same trend because they are estimated simultaneously. When the iteration number exceeds six, the RMSE of the frequency and phase is almost unchanged. So considering both the computation burden and tracking accuracy, the number of iteration is set to six in the following simulations. 

### 5.2. Simulation Results of ML-KF Loop

To demonstrate the effectiveness of the proposed adaptive KF, two simulations in two dynamic scenarios, pedestrian-level and vehicle-level, are made. The pedestrian-level velocity is expressed as 3sin(0.6t) m/s with a velocity, acceleration and acceleration rate that can reach a of maximum 3 m/s, 1.8 m/s^2^ and 1.08 m/s^3^, respectively. The vehicle-level velocity is expressed as 30sin(0.3t), with a velocity, acceleration and acceleration rate that can reach the maximum 30 m/s, 9 m/s^2^ and 2.7 m/s^3^, respectively. Considering the maximum acceleration (9 m/s^2^), the Doppler change over the integration time is only 0.047 Hz. Similarly, the phase change caused by the Doppler change over the integration time is only 0.0003 rad. Therefore, the assumption of constant Doppler frequency and phase over the integration time is still valid. *C*/*N*_0_ is 25 dB-Hz. In the ML-KF loop, $q_{a}$ is set to 1.8 and 9 in these two dynamic scenarios, respectively, which are equal to the maximum acceleration. The other parameters are the same as those in Table 1. The proposed MLE carrier tracking loop with and without KF is abbreviated as ML-KF and ML loop, respectively.

**Figure 8:**
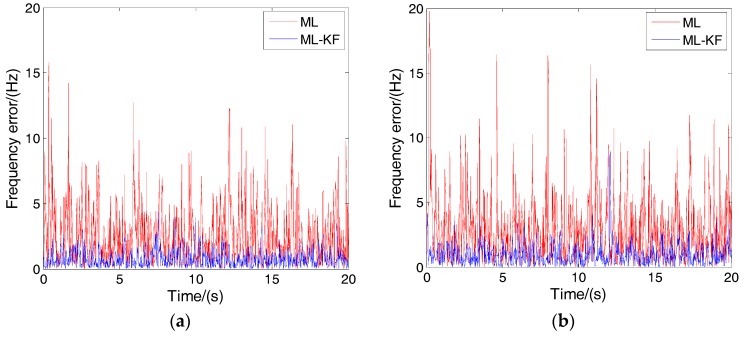
Tracking results of the ML (maximum likelihood) and ML-KF loops in different dynamic circumstances. (**a**) Frequency tracking results of pedestrian-level velocity; (**b**) Frequency tracking results of vehicle-level velocity.

Figure 8a,b shows the frequency tracking errors of these two loops in pedestrian and vehicle level scenarios, respectively. It can be seen that the errors of the ML-KF loop are smaller than that of the ML loop in two dynamic scenarios. The tracking accuracy is improved significantly by KF. For the ML-KF loop, the bit error rate is 0.001 and 0.002, respectively in pedestrian- and vehicle-level scenarios. For the ML loop, the bit error rate is 0.013 and 0.006 respectively in pedestrian and vehicle level scenarios. This means the bit error rate is reduced by filtering too. Therefore, the proposed KF can improve the tracking accuracy and reduce the bit error rate. 

### 5.3. Monte Carlo Simulations for Sensitivity and Accuracy

To provide intuitive quantification analysis, MC simulations are performed in terms of the sensitivity, accuracy and bit error rate of the proposed loop. A classic two-order FLL-assisted three-order PLL (FPLL) is also simulated to make a comparison [19]. This loop integrates both the FLL and the PLL characteristics. It is designed to satisfy the need for both the dynamic robustness of FLL plus the accuracy performance of the PLL. The discriminator algorithms of two-order FLL and three-order PLL are shown in Table 3. In order to keep consistent with the proposed loops, the integration time of PLL is set to 20 ms. Because the discriminator of FLL uses two integration results in an update, its integration time is set to 10 ms. The bandwidth of FLL and PLL is 4 Hz and 18 Hz, respectively, which is the optimal choice for low and high dynamics [19]. The simulation time is 10 s and 200 MC simulations are made. The other parameters are the same as those in Table 1. 

**Figure 9:**
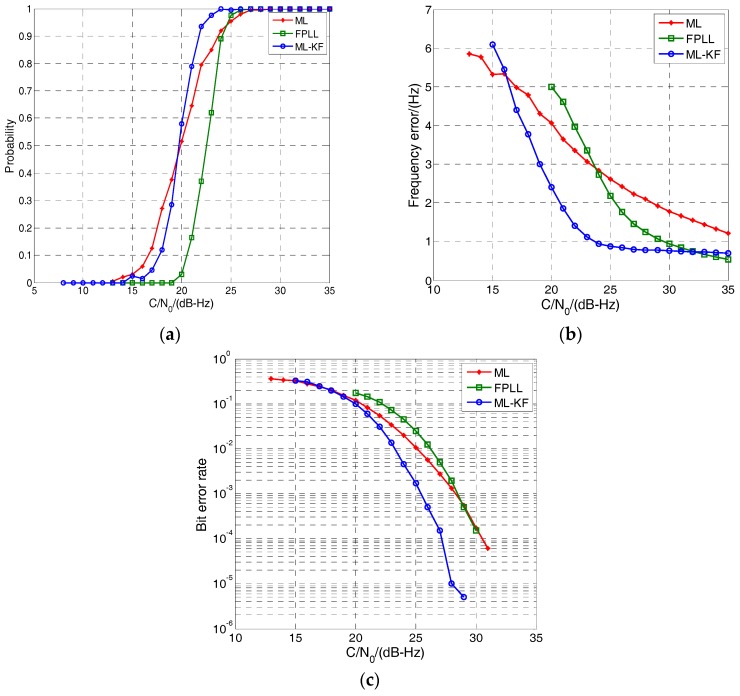
Performance comparison in pedestrian-level dynamic circumstances. (**a**) Tracking probability; (**b**) Frequency error; (**c**) Bit error rate. FPLL: two-order FLL-assisted three-order PLL.

The tracking performance of the proposed loop and FPLL in pedestrian-level dynamic circumstances is shown in Figure 9. The tracking probability is the ratio of successful tracking to Monte Carlo simulations. A successful tracking means that 1-sigma frequency error is less than 5 Hz and the maximum frequency error is less than 20 Hz during 10 s. The sensitivity is defined as the C/N0 where the tracking probability reaches 50%. 

It can be seen from Figure 9a that the sensitivity of the ML-KF and ML loop is approximately 19.5 dB-Hz, which is 3 dB-Hz lower than FPLL (22.5 dB-Hz). Therefore, the proposed MLE-based loops have lower *C*/*N*_0_ tracking threshold than the conventional FPLL. The frequency errors are shown in Figure 9b. The ML-KF loop shows the highest accuracy when *C*/*N*_0_ is below 32 dB-Hz. In contrast, the accuracy of the ML loop is much lower due to the lack of filtering. FPLL shows high accuracy when the *C*/*N*_0_ is above 30 dB-Hz. However, its performance decreases rapidly with *C*/*N*_0_ decreases. Figure 9c shows the bit error rate. It can be seen that the ML-KF loop has an approximately 8% error rate reduction compared with conventional FPLL. In summary, the ML-KF loop shows the optimal performance in sensitivity, precision and bit error rate in the weak signal and pedestrian-level dynamic circumstance.

**Figure 10:**
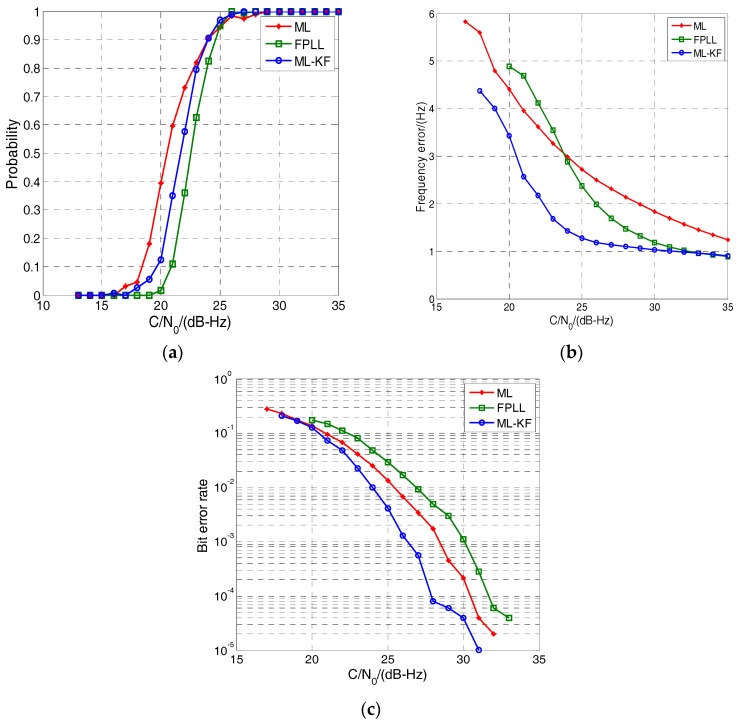
Performance comparison in vehicle-level dynamic circumstances. (**a**) Tracking probability; (**b**) Frequency error; (**c**) Bit error rate.

Figure 10 is similar to Figure 9 and shows the tracking performance comparison in the weak signal and vehicle-level dynamic circumstance. It can be seen from Figure 10a that the sensitivity of the ML-KF and ML loop reduces by 2.5 dB and 1 dB, respectively. The ML loop reaches to the lowest *C*/*N*_0_ tracking threshold. In contrast, the sensitivity of the conventional FPLL seems almost unchanged because two-order FLL can provide dynamic assistant for PLL. The frequency errors and bit error rate of all the three loops have a growing trend compared with Figure 9. However, the ML-KF loop still can provide the highest tracking accuracy and the lowest bit error rate. Therefore, the ML-KF loop still has better performance than the conventional FPLL in terms of sensitivity, accuracy and bit error rate.

In summary, the proposed ML-KF loop can reach the better performance than the conventional FPLL in terms of sensitivity, accuracy and bit error rate in weak signal and low dynamic circumstances. It is suitable for some land applications such as indoor pedestrian navigation and vehicle navigation. 

### 5.4. Optimal Loop Design

As analyzed above, the ML-KF loop seems to have no advantage over the FPLL when the *C*/*N*_0_ is above 30 dB-Hz. To reduce the computation burden and achieve the optimal performance, a switchable loop is designed to switch between the ML-KF loop and FPLL. When the *C*/*N*_0_ is above 30 dB-Hz, the loop works in FPLL. When the *C*/*N*_0_ is below 30 dB-Hz, the loop switches to the ML-KF loop. The *C*/*N*_0_ of FPLL is estimated by a conventional power ratio method (PRM) [20]. 

**Figure 11:**
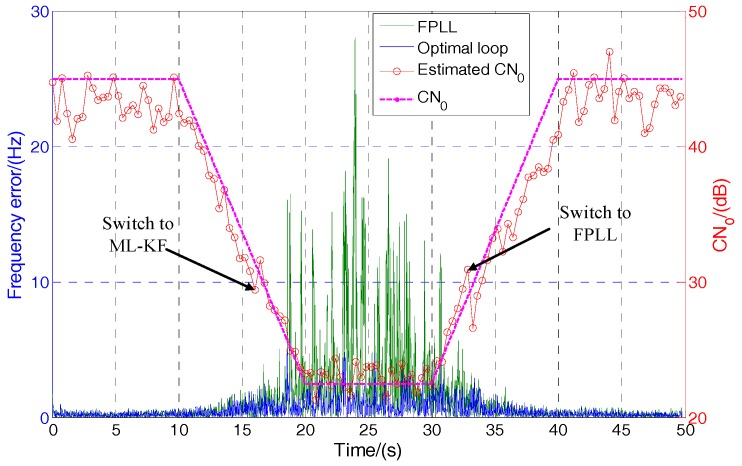
Performance comparison between the optimal loop and FPLL.

Figure 11 shows the performance comparison between the optimal loop and FPLL. The *C*/*N*_0_ of ML-KF loop is calculated by (8) and (29) and outputs at a rate of 50 Hz. To improve the estimation accuracy, estimation values are averaged in groups of 20. Therefore, the actual output rate of *C*/*N*_0_ is 2.5 Hz. The receiver moves at the vehicle-level velocity. The initial *C*/*N*_0_ is 45 dB-Hz. It drops to 22.5 dB-Hz form 10 s to 20 s. Then, it rises to 45 dB-Hz again from 30 s to 40 s. The frequency errors of the optimal loop and single FPLL are compared. It can be seen from Figure 11 that the FPLL outputs big errors when *C*/*N*_0_ is low. However, the optimal loop switches to ML-KF loop at 16.2 s and switches to FPLL at 33.2 s. Therefore, it can maintain high accuracy during the whole process. It can be seen from the estimated *C*/*N*_0_ curve that the proposed *C*/*N*_0_ estimation method is also effective.

## 6. Conclusions

A low-complexity MLE-based carrier tracking loop is presented in this paper. It is designed to work in some low dynamic and weak signal circumstances such as indoor pedestrian and urban vehicle navigation. Compared with the conventional MLE-based methods, this method is operated based on the coherent integration results instead of the sampling data, which reduces the computation burden significantly. The MLE discriminator is designed and its performance is improved by an adaptive KF. Compared with the conventional two-order FLL assisted three-order PLL, its sensitivity has an improvement of 3 dB and 1 dB in pedestrian-level and vehicle-level dynamic circumstances, respectively. It also shows higher accuracy and lower bit error rate than the conventional loop. It is suitable for indoor pedestrian and urban vehicle navigation.

## Figures and Tables

**Table 1:** Dynamic tolerance and signal-to-noise ratio (SNR) gain of the proposed algorithm.

**Discriminator Update Time (ms)**	20	40	60	80	100	200
**Dynamic Tolerance (m/s^2^)**	357	44.6	19.8	11.1	7.1	1.8
**SNR Gain (dB)**	76.1	72.9	74.7	75.9	76.9	79.9

**Table 2:** Simulation parameters.

Parameter	Value
Carrier-to-noise ratio *C*/*N*_0_	28 dB-Hz
Sampling rate *f_s_*	5.714 MHz
Integration time *T*	1 ms
Observation point number *N*	20
Iteration number threshold *M*	20
Gradient threshold Γ	0.01

**Table 3:** Discriminator algorithms of two-order frequency-locked loop (FLL) and three-order phase-locked loop (PLL).

	Discriminator Algorithm
**Two-order FLL**	$\frac{ATAN2(cross,dot)}{2\pi T}$ where ATAN2(∗) denotes four-quadrant arctangent, $dot=R(r_{n-1})R(r_{n})+I(r_{n-1})I(r_{n})$, $cross=R(r_{n-1})I(r_{n})-R(r_{n})I(r_{n-1})$
**Three-order PLL**	$ATAN\left(\frac{I(r_{n})}{Q(r_{n})}\right)$ where ATAN(∗) denotes the two-quadrant arctangent.
